# Safety and Efficacy of Dolutegravir in Treatment-Experienced Subjects With Raltegravir-Resistant HIV Type 1 Infection: 24-Week Results of the VIKING Study

**DOI:** 10.1093/infdis/jis750

**Published:** 2012-12-07

**Authors:** Joseph J. Eron, Bonaventura Clotet, Jacques Durant, Christine Katlama, Princy Kumar, Adriano Lazzarin, Isabelle Poizot-Martin, Gary Richmond, Vincent Soriano, Mounir Ait-Khaled, Tamio Fujiwara, Jenny Huang, Sherene Min, Cindy Vavro, Jane Yeo, for the VIKING Study Group, Sharon L. Walmsley, Joseph Cox, Jacques Reynes, Philippe Morlat, Daniel Vittecoq, Jean-Michel Livrozet, Pompeyo Viciana Fernández, Jose M. Gatell, Edwin DeJesus, Jerome DeVente, Jacob P. Lalezari, Lewis H. McCurdy, Louis A. Sloan, Benjamin Young, Anthony LaMarca, Trevor Hawkins

**Affiliations:** 1 Infectious Diseases Division, School of Medicine, University of North Carolina, Chapel Hill; 2 GlaxoSmithKline, Research Triangle Park, North Carolina; 3 Georgetown University, Washington, D.C.; 4 Broward Health, Fort Lauderdale, Florida; 5 Hospital Germans Trias i Pujol, Irsicaixa Foundation, Barcelona, France; 6 Hospital Carlos III, Madrid, Spain; 7 Hôpital de l'Archet 1, Nice, France; 8 Hôpital de la Pitié-Salpêtrière, Paris VI University, Paris, France; 9 APHM Sainte-Marguerite, Aix Marseille University, Marseille, France; 10 San Raffaele Scientific Institute, Milan, Italy; 11 GlaxoSmithKline, London, United Kingdom; 12 Shionogi & Co Ltd, Osaka, Japan; 13 GlaxoSmithKline, Toronto, Canada

**Keywords:** Dolutegravir, DTG, S/GSK1349572, integrase inhibitor, raltegravir resistance

## Abstract

***Background.*** Dolutegravir (DTG; S/GSK1349572), a human immunodeficiency virus type 1 (HIV-1) integrase inhibitor, has limited cross-resistance to raltegravir (RAL) and elvitegravir in vitro. This phase IIb study assessed the activity of DTG in HIV-1–infected subjects with genotypic evidence of RAL resistance.

***Methods.*** Subjects received DTG 50 mg once daily (cohort I) or 50 mg twice daily (cohort II) while continuing a failing regimen (without RAL) through day 10, after which the background regimen was optimized, when feasible, for cohort I, and at least 1 fully active drug was mandated for cohort II. The primary efficacy end point was the proportion of subjects on day 11 in whom the plasma HIV-1 RNA load decreased by ≥0.7 log_10_ copies/mL from baseline or was <400 copies/mL.

***Results.*** A rapid antiviral response was observed. More subjects achieved the primary end point in cohort II (23 of 24 [96%]), compared with cohort I (21 of 27 [78%]) at day 11. At week 24, 41% and 75% of subjects had an HIV-1 RNA load of <50 copies/mL in cohorts I and II, respectively. Further integrase genotypic evolution was uncommon. Dolutegravir had a good, similar safety profile with each dosing regimen.

***Conclusion.*** Dolutegravir 50 mg twice daily with an optimized background provided greater and more durable benefit than the once-daily regimen. These data are the first clinical demonstration of the activity of any integrase inhibitor in subjects with HIV-1 resistant to RAL.

Integrase inhibitors (INIs) represent a class of drugs for the treatment of human immunodeficiency virus (HIV)–infected individuals, blocking HIV genome integration into the host cell DNA [1]. They have been shown to be highly effective for the treatment of antiretroviral-naive and antiretroviral-experienced subjects, as demonstrated first with raltegravir (RAL) and more recently with elvitegravir (EVG) [2–6]. However, these first-generation INIs share common resistance pathways. In clinical studies of RAL, subjects with virologic failure and reduced RAL susceptibility typically harbored virus with 1 of 3 signature mutational pathways (ie, N155H, Q148H/K/R, or Y143C/H/R) in the integrase gene [7]. Continuing RAL treatment in these circumstances may lead to the addition of secondary mutations or pathway evolution; N155H may evolve to Y143 or Q148 pathways [4]. In addition, EVG does not appear to have activity against RAL-resistant isolates, and RAL does not appear to have activity against EVG-resistant isolates [8–10]. Therefore, there is a need for an INI with a high barrier to resistance and activity in subjects with human immunodeficiency virus type 1 (HIV-1) resistant to EVG and RAL.

Dolutegravir (DTG) is a new HIV-1 INI that has demonstrated good efficacy and safety in treatment-naive, HIV-infected individuals [11]. In vitro studies demonstrate limited cross-resistance between DTG and RAL or EVG, with no or minimal impact on DTG fold-change against Q148 single mutants or against viruses with Y143 or N155 signature mutations regardless of RAL-associated secondary mutations [12, 13]. However, the DTG fold-change increased for Q148H/K/R as secondary RAL resistance–associated mutations increased. On the basis of these in vitro findings, this phase IIb pilot study was conducted to assess and demonstrate the activity of DTG in HIV-1–infected individuals with RAL-resistant viral isolates.

## METHODS

### Study Design

VIKING (ING112961) is a phase IIb, multicenter, open-label, single-arm, pilot study with 2 sequential cohorts of HIV-1–infected individuals with current or historic RAL treatment failure and evidence of RAL resistance at screening. The 50-mg once-daily dose of DTG was initially selected for evaluation (cohort I); however, the viral load response of some subjects prompted protocol amendment and subsequent evaluation of DTG 50 mg as a twice-daily regimen (cohort II). For inclusion in cohort I, subjects were screened from August 2009 to October 2009; for inclusion in cohort II, subjects were screened from June 2010 to October 2010.

**Figure 1.**
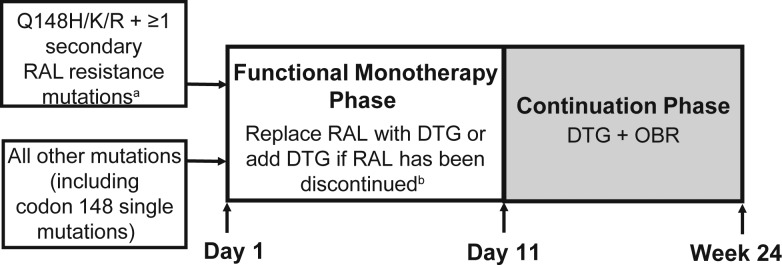
Study design. Subjects received dolutegravir (DTG) 50 mg once daily (cohort I) or DTG 50 mg twice daily (cohort II). In both cohorts, subjects were allocated to 1 of 2 groups on the basis of integrase genotype at screening, to ensure a broad representation of genotypes and a range of phenotypic susceptibility. Cohort II subjects were required to have ≥1 fully active antiretroviral therapy in an optimized background regimen (OBR). Abbreviation: RAL, raltegravir. ^a^Q148H/K/R plus changes in L74, E138, or G140. ^b^Subjects remained on failing background regimen from day 1 to day 10.

The study treatment phases for both cohorts consisted of an initial 10-day period, when DTG was administered with the failing background regimen (RAL was discontinued prior to DTG dosing), followed by a second phase (day 11 onward), when DTG therapy was maintained but the background therapy could be optimized according to genotypic and phenotypic tests. Subjects remained in the study through at least 24 weeks if they were receiving a clinical benefit from participating. Subjects were allocated at screening to 1 of 2 groups on the basis of integrase genotype, as specified in Figure 1, with capping, as appropriate, to ensure balanced distribution of subjects between the 2 groups. The 2 groups were not used for analysis; their purpose was to secure a broad range of DTG sensitivity in the enrolled population.

Study visits were conducted at screening; on days 1, 6–8, 10, 11, and 21; and at weeks 4, 8, 12, 16, 20, and 24. The study was conducted in France, Italy, Canada, Spain, and the United States and was approved by the respective regulatory authorities in each country and by the ethics review committee associated with each of the 25 study sites. Written informed consent was obtained from each individual before screening. Protocol summaries are posted online (http://www.clinicaltrials.gov [clinical trials registration NCT00950859] and http://www.gsk-clinicalstudyregister.com [112961]).

### Subjects

Antiretroviral therapy (ART)–experienced, HIV-1–infected adults (≥18 years of age) with plasma HIV-1 RNA levels of ≥1000 copies/mL, genotypic INI resistance, and documented genotypic and/or phenotypic resistance to ≥1 compound in each of 2 other approved classes of ART (nucleoside reverse transcriptase inhibitors [NRTIs], nonnucleoside reverse transcriptase inhibitors [NNRTIs], protease inhibitors [PIs], and fusion/entry inhibitors) were eligible for enrollment. The availability of ≥1 fully active ART agent for the optimized background regimen was encouraged for cohort I but mandated for cohort II eligibility. Exclusion criteria included preexisting mental, physical, or substance abuse disorders that could interfere with study conduct; a screening alanine aminotransferase concentration of ≥5 times the upper limit of normal; or a screening lipase concentration of ≥3 times the upper limit of normal. Pregnant or breast-feeding women were excluded. Given available data on drug-drug interactions, subjects were not enrolled if they were currently receiving or anticipated requiring efavirenz, nevirapine, fosamprenavir/ritonavir, or tipranavir/ritonavir. Etravirine (ETR) was required to be coadministered with lopinavir/ritonavir or darunavir/ritonavir (DRV/r) within 15 days of DTG dosing and/or during the study, to avoid subject exclusion.

### Efficacy Assessments

The primary efficacy end point was the proportion of subjects on day 11 with a plasma HIV-1 RNA load of <400 copies/mL or of ≥0.7 log_10_ copies/mL below the baseline value, as quantified by the Abbott RealTime HIV-1 amplification assay (Abbott Molecular, Des Plaines, IL). Secondary efficacy end points included at day 11 and subsequent visits were as follows: mean change from baseline in plasma HIV-1 RNA level; proportion of subjects with plasma HIV-1 RNA loads of <400 and <50 copies/mL, on the basis of the time to loss of virologic response algorithm; and change from baseline CD4^+^ T-cell count.

Protocol-defined virologic failure was defined in relation to baseline plasma HIV-1 RNA levels, as follows: at day 11, a decrease of <0.7 log_10_ copies/mL, unless a level of <400 copies/mL was achieved; at weeks 8 to <16, a decrease of <1.0 log_10_ copies/mL, unless a level of <400 copies/mL was achieved, or an increase of ≥1.0 log_10_ copies/mL from the nadir level; and at weeks ≥16, a level of ≥400 copies/mL.

### Safety Assessments

Safety parameters, including adverse events (AEs), laboratory parameters, electrocardiogram findings, and vital signs, were evaluated at scheduled time points and when clinically indicated. The severity of AEs and laboratory abnormalities was reported and assessed using the grading table of the Division of Acquired Immunodeficiency Syndrome, National Institute of Allergy and Infectious Diseases [14]. All available safety data as of the week 24 data cutoff for each cohort are presented here.

### Viral Genotyping and Phenotyping Assessments

Genotypic and phenotypic assays were performed on plasma samples by Monogram Biosciences (South San Francisco, CA), using PhenoSense and GenSeq testing methods for NRTIs, NNRTIs, PIs, and INIs; PhenoSense Entry testing for enfuvirtide (T-20) susceptibility; and the Trofile assay for maraviroc (MVC) susceptibility. Phenotypic susceptibility was reported as the fold-change relative to that for wild-type virus. The phenotypic susceptibility score is the total number of ART drugs in the background regimen to which a subject's HIV is fully susceptible on the basis of phenotypic testing. Baseline integrase genotypic resistance data were used to produce 6 categories for analysis that were based on mutational pathway: (1) Q148 + 1 (associated mutation at 1 of codons 74, 138, or 140 but not at codons 155 or 143); (2) Q148 + 2 (associated mutations at 2 of codons 74, 138, or 140 but not at codons 155 or 143); (3) mixture (mutations at codons 148, 155, or 143); (4) N155 (mutations at codon 155 but not at codons 148 or 143); (5) Y143 (mutations at codon 143 but not at codons 148 or 155); and (6) other (no mutations at codons 148, 155, or 143). A comparison of on-treatment to day 1 genotypic and phenotypic data was made for all subjects with data available at day 11 (only samples with a plasma HIV-1 RNA level of ≥150 copies/mL were tested) and for subjects meeting the criteria for protocol-defined virologic failure.

**Figure 2.**
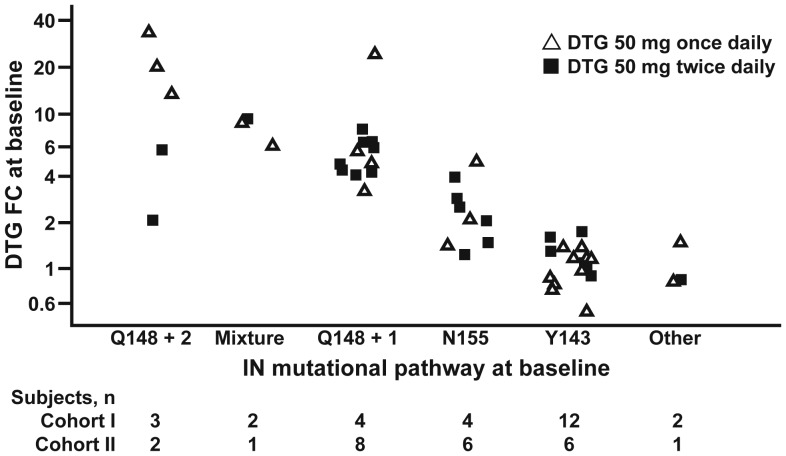
Dolutegravir (DTG) fold-change (FC) in 50% inhibitory concentration at baseline, by integrase (IN) mutational pathway at baseline.

### Statistical Analysis

A single-arm cohort with 30 subjects (cohort I) has approximately 90% power to distinguish between a failure rate of 10% and ≥40% at a 2-sided α level of 0.05, while a single-arm cohort with 20 subjects (cohort II) has approximately 80% power to detect such a difference. The intent-to-treat exposed population consisted of subjects who received ≥1 dose of DTG and who had ≥1 on-treatment measure of plasma HIV-1 RNA level. Baseline characteristics and antiviral activity, by cohort, were summarized using descriptive statistics. The last observation carried forward and discontinuation equals baseline data set was used for the primary efficacy analysis, change from baseline in CD4^+^ T-cell count, and HIV-1 RNA level at week 24. The assessment of response at week 24 below a fixed threshold was calculated according to the US Food and Drug Administration's time to loss of virologic response algorithm [15].

The antiviral activity on day 11 between the 2 cohorts was compared using a linear regression model, with adjustment for baseline viral load, DTG fold-change relative to wild-type virus at baseline, phenotypic susceptibility score of the failing background regimen (days 1–10), and the integrase mutational pathway at baseline. The population evaluated for safety included all subjects who received ≥1 dose of DTG.

## RESULTS

### Demographic and Baseline Characteristics

Of 53 and 54 subjects screened for cohorts I and II, respectively, 27 and 24 subjects were enrolled to compose the intent-to-treat exposed populations. Demographic and baseline characteristics generally were similar for the 2 cohorts (Table 1). Subjects were predominantly white men, with a median age of 48 years. Twenty-six percent and 29% of the subjects in cohorts I and II, respectively, had advanced HIV disease, defined on the basis of a CD4^+^ T-cell count of <50 cells/mm^3^. Both cohorts had extensive previous ART treatment, although more subjects in cohort I had received ETR, DRV/r, and/or T-20. Because, by design, cohort II subjects should have received ≥1 fully active optimized background regimen component from day 11, only 1 cohort II subject (4%) had an optimized background regimen phenotypic susceptibility score of 0, compared with 12 subjects (44%) in cohort I (treatment for this subject was not a protocol violation because the activity of the fully active background agent was lost between screening and baseline). Baseline resistance data indicated the most common fully active optimized background regimen components to be tenofovir (n = 9), ETR (n = 5), DRV/r (n = 4), MVC (n = 2), and T-20 (n = 2) for cohort I and DRV/r (n = 11), ETR (n = 9), tenofovir (n = 7), MVC (n = 4), and T-20 (n = 3) for cohort II.

**Table 1.** Demographic and Baseline Characteristics for the Intent-to-Treat Exposed Population

Parameter	Cohort I, DTG 50 mg Once Daily (n = 27)	Cohort II, DTG 50 mg Twice Daily (n = 24)
Age, y	48 (19–61)	47 (33–68)
Male sex	25 (93)	18 (75)
Race		
White, Caucasian/European heritage	23 (85)	18 (75)
White, Arabic/North African heritage	1 (4)	1 (4)
African American/African heritage	3 (11)	5 (21)
CD4^+^ T-cell count, cells/mm^3^	114 (19–729)	202 (19–528)
Plasma HIV-1 RNA level, log_10_ copies/mL	4.5 (2.6–6.1)	4.3 (3.3–5.8)
CDC class C disease	16 (59)	8 (33)
Hepatitis virus coinfection		
HBsAg positive	0	2 (9)
HCV antibody positive	2 (7)	6 (26)
HBV or HCV status missing	4 (15)	2 (9)
HBsAg and HCV antibody positive	0	0
Duration of prior ART, y	14 (4–21)	15 (3–22)
No. of prior ART drugs	17 (6–24)	15 (6–19)
Prior ART treatment		
Etravirine	19 (70)	11 (46)
Enfuvirtide	22 (81)	13 (54)
Darunavir/ritonavir	23 (85)	14 (58)
Maraviroc	10 (37)	9 (38)
Current RAL failure	21 (78)	20 (83)
Baseline INI resistance/integrase mutational pathway		
Q148H/K/R + 2 mutations^a^	3 (11)	2 (8)
Q148H/K/R + 1 mutations^a^	4 (15)	8 (33)
Mixed Q148H/K/R with Y143C/H/R or N155H	2 (7)	1 (4)
N155H	4 (15)	6 (25)
Y143C/H/R	12 (44)	6 (25)
Other^b^	2 (7)	1 (4)
Baseline DTG FC	1.46 (0.55–35)	2.72 (0.87–9.48)
Baseline RAL FC	>161 (0.6–166)	>128 (0.8–183)
PSS of failing regimen = 0^c^	18 (67)	15 (63)
PSS of OBR = 0 (on day 11)	12 (44)	1 (4)

Data are no. (%) of subjects or median (range).

Abbreviations: ART, antiretroviral therapy; CDC, Centers for Disease Control and Prevention; DTG, dolutegravir; FC, fold change in 50% inhibitory concentration; HBsAg, hepatitis B virus surface antigen; HBV, hepatitis B virus; HCV, hepatitis C virus; INI, integrase inhibitor; OBR, optimized background regimen; PSS, phenotypic susceptibility score; RAL, raltegravir.

^a^ Mutations at L74, E138, or G140.

^b^ Subjects with no Q148, Y143, or N155 mutations at day 1.

^c^ PSS on fully active agents.

The median baseline RAL fold-change was greater than the maximum concentration tested for viruses isolated from both cohorts I and II (Table 1). In contrast, the median fold-change for DTG was low (cohort I, 1.46; cohort II, 2.72), with low-to-moderate values for Y143 and N155H pathway viruses (Figure 2) but higher values for viruses with mutations at Q148.

### Efficacy

Ninety-six percent of subjects (23 of 24) in cohort II and 78% of subjects (21 of 27) in cohort I achieved the primary end point (ie, a reduction in plasma HIV-1 RNA level of ≥0.7 log_10_ copies/mL below the baseline value or a level of <400 copies/mL at day 11; Table 2). Thirteen subjects (54%) in cohort II and 11 subjects (41%) in cohort I achieved an HIV-1 RNA level of <400 copies/mL on day 11. The respective numbers for subjects with HIV-1 RNA levels of <50 copies/mL were 4 (17%) and 3 (11%). In a multivariate analysis that controlled for baseline factors, cohort II had a significantly larger reduction in HIV-1 RNA level from baseline on day 11, compared with cohort I (mean adjusted treatment difference, −0.32 log_10_ copies/mL; −1.76 vs −1.45 log_10_ copies/mL; *P* = .017).

**Table 2.** Efficacy Results for the Intent-to-Treat Exposed Population

Variable	Cohort I, DTG 50 mg Once Daily (n = 27)	Cohort II, DTG 50 mg Twice Daily (n = 24)
Efficacy at day 11		
Primary end point, no. (%)	21 (78)	23 (96)
Plasma HIV-1 RNA level, log_10_ copies/mL		
Baseline, mean (SD)	4.40 (0.79)	4.38 (0.74)
Day 11, mean (SD)	2.94 (1.01)	2.62 (0.78)
Change from baseline, mean (SD)	−1.45 (0.77)	−1.76 (0.54)
Model-adjusted change, mean (SD)	−1.45 (0.08)	−1.76 (0.09)
Adjusted treatment difference, mean (95% CI)^a^	−0.32 (−0.57 to −0.06)^b^	
Efficacy at week 24		
HIV-1 RNA load, copies/mL, no. (%)^c^		
<50	11 (41)	18 (75)
<400	14 (52)	20 (83)
<50, by baseline PSS to OBR at day 11		
PSS = 0	1/12 (8)	1/1 (100)
PSS = 1	4/7 (57)	6/9 (67)
PSS ≥ 2	6/8 (75)	11/14 (79)
HIV-1 RNA load <50 copies/mL, no. (%)^c^		
Responder	11 (41)	18 (75)
Virologic failure	13 (48)	5 (21)
Never suppressed or discontinued for insufficient viral load response	11 (41)	4 (16)
Rebound	2 (7)	1 (4)
Discontinued study drug or added new ART before achieving confirmed suppression	3 (11)	1 (4)
AE/death	2 (7)^d^	0
Nonpermitted change in ART	1 (4)	1 (4)
HIV-1 RNA level, mean change from baseline (SD), log_10_ copies/mL^e^	−1.3 (1.29)	−2.3 (1.05)
Change in CD4^+^ T-cell from baseline, cells/ mm^3^, median (IQR)^e^	54.0 (0–118.0)	60.0 (0–145.5)

Abbreviations: AE, adverse event; ART, antiretroviral therapy; CI, confidence interval; DTG, dolutegravir; IQR, interquartile range; LOCFDB, last observation carried forward and discontinuation equals baseline; OBR, optimized background regimen; PSS, phenotypic susceptibility score; TLOVR, time to loss of virologic response.

^a^ Cohort II vs cohort I.

^b^ *P* = .017.

^c^ Based on TLOVR algorithm.

^d^ One subject with brain mass subsequently died. One subject with febrile bone marrow aplasia died on the last date of DTG administration.

^e^ Analysis based on LOCFDB data set at week 24 visit.

At week 24, the response rate was greater in cohort II: 18 subjects (75%) had a plasma HIV-1 RNA level of <50 copies/mL, in contrast to 11 subjects (41%) in cohort I (Table 2). The response rates increased in both cohorts as the number of fully active agents in the optimized background regimen increased. The CD4^+^ T-cell count increased from baseline in both cohorts through week 24 (median increase, 54 and 60 cells/mm^3^ for cohorts I and II, respectively).

New HIV-associated conditions were reported for 4 subjects in cohort I and 1 subject in cohort II through week 24. These conditions included oropharyngeal candidiasis, herpes simplex virus infection, immunoblastic lymphoma, and a brain mass in cohort I and oropharyngeal candidiasis in cohort II.

### Safety

The safety profile of DTG was similar in both cohorts. Adverse events (grade ≥2) were reported by 13 subjects (48%) and 16 subjects (67%) in cohorts I and II, respectively (Table 3), but no obvious trend in increased reporting of any individual parameter was observed in cohort II. Serious AEs were reported in 4 and 3 subjects in cohorts I and II, respectively; none of these were considered related to DTG treatment, and no specific serious AE was reported by >1 subject. Two deaths were reported in cohort I: 1 subject with an immunoblastic lymphoma/bone marrow aplasia died during a second course of chemotherapy, and 1 subject died, after study withdrawal, with an undiagnosed brain mass. There were no other study discontinuations due to AEs in cohort I. In cohort II, there were no deaths or discontinuations due to AEs.

**Table 3.** Summary of Grade ≥2 Adverse Events

Grade ≥2 AE	Cohort I, DTG 50 mg Once Daily (n = 27)	Cohort II, DTG 50 mg Twice Daily (n = 24)
Any	13 (48)	16 (67)
Diarrhea	1 (4)	2 (8)
Insomnia	3 (11)	0
Bronchitis	1 (4)	2 (8)
Cough	1 (4)	2 (8)

Data are no. (%) of subjects and are limited to grade ≥2 AEs occurring in ≥2 subjects in cohort I or cohort II.

Abbreviations: AE, adverse event; DTG, dolutegravir.

The proportion of grade 3 treatment-emergent laboratory abnormalities was similar between cohorts (cohort I, 22%; cohort II, 21%), and only 1 subject in cohort II experienced a grade 4 laboratory abnormality (neutropenia). Of the grade 3 abnormalities, elevated total and low-density lipoprotein cholesterol, lipase, and bilirubin levels and decreased phosphorus level were each reported for 2 subjects in both cohorts combined. In both cohorts, modest mean increases in creatinine concentration were noted; increases were evident by days 6–8 (mean increase, 0.084–0.105 mg/dL), reached a plateau at week 4 (mean increase, approximately 0.14 mg/dL for both cohorts), and remained stable to week 24. No subject had a grade 3 or 4 increase in creatinine values, and renal toxicity did not result in withdrawal of any subjects.

### Virologic Resistance

To assess the evolution of integrase resistance in the context of DTG coadministered with failing background therapy, 18 of 27 subjects in cohort I and 15 of 24 subjects in cohort II had a sufficient plasma HIV-1 RNA level (≥150 copies/mL) on day 11 for assessment. Viruses from only 2 of 18 subjects in cohort I and 3 of 15 subjects in cohort II demonstrated emergence of additional integrase resistance mutations, a reduction in DTG susceptibility, or both on day 11 (Table 4). Four of these 5 subjects harbored virus with Q148H plus ≥1 additional RAL resistance–associated mutation at day 1. Of these 4 subjects, 3 had an increase in DTG fold-change for the day 11 virus, while no phenotypic result was obtained for virus from the fourth subject. The fifth subject (S1) harbored a virus from the Y143R pathway at day 1 and, despite detection of an E138E/K mixture at day 11, DTG fold-change remained unchanged from baseline. All 3 subjects in cohort II had a phenotypic susceptibility score for their failing regimen of 0, and the 2 subjects in cohort I each had a phenotypic susceptibility score of 1. Both subjects in cohort I and 1 subject in cohort II were able to reach a plasma HIV-1 RNA level of <400 copies/mL after day 11.

**Table 4.** Summary of Subjects Demonstrating Integrase Genotype Evolution, Reduction in Dolutegravir (DTG) Susceptibility, or Both on Day 11^a^

Subject	Integrase Mutational Group	Change in HIV-1 RNA From Baseline, log_10_ Copies/mL	PSS of Failing Regimen on Day 1	RAL Resistance–Associated Mutations^b^		DTG FC	
				Day 1	Day 11	Day 1	Day 11
Cohort I							
S1	Y143	−1.61	1	L74M, T97A, Y143R, G163G/R	L74L/M, T97A, **E138E/K**, Y143Y/ R/H/C, G163G/R	1.03	1.09
S2	Mixture	−0.32	1	G140S, **Y143H**, Q148H	**L74I/M, E138E/A**, G140S, Q148H	6.49	38
Cohort II							
S3	Q148 + 2	−1.79	0	E138E/K, G140G/S, Q148Q/H	**T97T/A**, E138E/K, G140S, Q148H	2.1	11
S4	Q148 + 1	−1.57	0	G140S, Q148H	G140S, Q148H, **N155N/H**	6.23	NR
S5	Q148 + 2	−0.9	0	E138A, G140S, Q148H	**T97T/A**, E138T/A, G140S, Q148H	6.04	21

Abbreviations: FC, fold-change in 50% inhibitory concentration; NR, no results; PSS, phenotypic susceptibility score; RAL, raltegravir.

^a^ In subjects with paired day 1 and day 11 integrase genotype and phenotype.

^b^ Differences in integrase genotype between day 1 and day 11 are bold.

Protocol-defined virologic failure was observed through week 24 in 12 subjects (44%) and 5 subjects (21%) in cohorts I and II, respectively. Ten of the 12 cohort I subjects had an optimized background regimen phenotypic susceptibility score of 0, in contrast to none of the 5 cohort II subjects. Treatment-emergent INI genotypic resistance was observed in virus isolated at protocol-defined virologic failure for 4 of the 12 cohort I subjects and 3 of the 5 cohort II subjects, accompanied by an increase in DTG fold-change (Table 5). At virologic failure, all 7 subjects harbored ≥4 RAL resistance–associated mutations, and 5 of the 7 subjects (2 in cohort I; 3 in cohort II) had virus with Q148 + ≥1 RAL resistance–associated mutation at screening or baseline. Virus from 4 of the 7 subjects had emergent N155H detected at virologic failure; however, the impact of this acquisition on the absolute DTG fold-change at protocol-defined virologic failure was dependent on the starting pathway virus.

**Table 5.** Treatment-Emergent Integrase Inhibitor (INI) Resistance Mutations at the Time of Protocol-Defined Virologic Failure (PDVF)

Subject	Integrase Mutational Group	VF Visit	PSS	RAL Resistance–Associated Mutations**^a^**		DTG FC	
				Day 1	Time of PDVF	Day 1	Time of PDVF
Cohort I							
S2	Mixture	Day 11	1	G140S, **Y143H**, Q148H	**L74I/M, E138E/A**, G140S, Q148H	6.49	38
S6	Other	Week 8	0	None^b^	**L74L/M/I, T97A, G140S, Q148H**	0.87	68
S7	Y143	Week 24	0	L74M, T97A, E138A, Y143R	L74M, T97A, E138A, Y143R, **N155H**	0.77	6.58
S8	Y143	Week 24	0	L74M, T97A, Y143R	L74M, T97A, Y143R, **N155H**	0.91	8.44
Cohort II							
S4	Q148 + 1	Week 16	2	G140S, Q148H	**T97T/A, E138E/K**, G140S, Q148H, **N155H**	6.23	93
S5	Q148 + 2	Week 8	1	E138A, G140S, Q148H	**E92E/Q, T97T/A**, G140S, Q148H	6.04	42.32
S9	Q148 + 1	Week 8	4	G140S, Q148H	**E138E/K**, G140S, Q148H, **N155H**	4.11	63

Abbreviations: DTG, dolutegravir; FC, fold-change in 50% inhibitory concentration; PSS, phenotypic susceptibility score; RAL, raltegravir; VF, virologic failure.

^a^ Differences in integrase genotype between day 1 and time to PDVF are bold.

^b^ Subject harbored virus with G140G/S and Q148Q/H at screening.

## DISCUSSION

VIKING is the first study to explore DTG treatment of HIV-1–infected subjects who had experienced virologic failure during receipt of a RAL-containing regimen and had genotypic evidence of RAL resistance. Despite the presence of HIV-1 with a very high level of resistance to RAL, a rapid antiviral response was observed in both cohorts, with a better response rate for the primary end point at day 11 in cohort II (96%) than in cohort I (78%). In a linear regression model accounting for differences in baseline factors and phenotypic susceptibility score of the continued failing regimen, the reduction in plasma HIV-1 RNA level at day 11 was shown to be significantly greater (*P* = .017) in cohort II than in cohort I. The twice-daily 50-mg dose of DTG was assessed in cohort II because clinical pharmacology data with DTG 100 mg once daily had indicated a solubility limit to DTG and because pharmacokinetic/pharmacodynamic modeling had predicted better long-term antiviral effects with DTG 50 mg twice daily against RAL-resistant viruses with greater fold-change in susceptibility to DTG [16]. The results presented here support these assumptions.

This study included highly treatment-experienced populations with HIV-1 resistant to most approved ART drugs and to RAL, and although the baseline viral isolates generally had relatively low fold-change in DTG susceptibility, the range of susceptibility to DTG allowed an appropriate test of this new INI. The baseline phenotypic susceptibility to DTG was narrower in cohort II, with no viruses showing a fold-change of ≥10 in susceptibility to DTG when compared with cohort I. Given the relatively small sample size and the fact that 23 of 24 subjects in cohort II responded to DTG, a phenotypic cutoff for DTG activity could not be established.

At week 24, more subjects in cohort II than cohort I achieved plasma HIV-1 RNA levels of <50 and <400 copies/mL, consistent with the predicted better drug exposure and a more active optimized background regimen as mandated by the protocol. Considering only those subjects with an optimized background regimen phenotypic susceptibility score of 1, the proportion of subjects achieving <50 copies/mL at week 24 was higher in cohort II, compared with cohort I (67% vs 57%). In both cohorts, subjects experienced an immunological response, with a median increase in CD4^+^ T-cell count of 54–60 cells/mm^3^ by week 24.

DTG generally was well tolerated when administered at either 50 mg once daily or 50 mg twice daily, consistent with safety data from other treatment studies [11], and did not show a clinically significant difference in the safety profile between the 2 dosages, although the sample size was small. Reporting rates for laboratory abnormalities were low and comparable across both cohorts. The modest, early, nonprogressive effect on creatinine level is consistent with inhibition of a renal transporter, organic cation transporter 2, as discussed in prior reports on DTG [11].

Resistance emergence during the initial 10-day phase was limited, with new INI resistance mutations detected in day 11 samples from 2 of 18 cohort I subjects and 3 of 15 cohort II subjects. Treatment-emergent genotypic resistance was observed in virus at protocol-defined virologic failure at or after day 11 from 4 of 12 cohort I subjects and from 3 of 5 cohort II subjects. All of these subjects harbored 2 or 3 INI resistance mutations at screening or baseline. Across both cohorts, virus with Q148H + G140S plus additional RAL resistance mutations was more likely to have a greater DTG fold-change and lower response to DTG treatment. These results are consistent with the earlier in vitro findings that mutations at position Q148 with additional INI resistance mutations can reduce DTG susceptibility [17]. For 2 subjects in cohort I (both with the Y143 mutation) and 2 subjects in cohort II (both with Q148 + 1 mutations), the addition of the N155H mutation to the resistance profile was observed, and, in each case, 3–4 RAL resistance mutations were present with N155H. Therefore, a substantial number of well-characterized INI resistance mutations appear to be required to confer reduced susceptibility to DTG in virus from patients previously treated with RAL with well-documented virologic failure. In this study, at all positions in the integrase gene that were evaluated, no DTG-specific resistance mutations have been identified thus far. Clonal analysis and site-directed mutant phenotypic analysis of observed mutations are ongoing to better define the role of these mutations on DTG phenotypic susceptibility and will be the subject of future analyses. A clonal analysis of the 4 samples in which the N155H mutation emerged at the time of virological failure showed that the emergent N155H mutation and the integrase resistance mutation detected at day 1 were on the same genome [18, 19]. This result provides further support for the conclusion that high-level DTG resistance requires multiple RAL resistance mutations. The possibility that the N155H mutation was present as a low-level minority variant at baseline, which has been observed in other studies of virological failure of first-generation INI [20], cannot be excluded.

The sequential cohort design, with some differences in baseline characteristics between cohorts and the mandate of cohort II subjects to receive ≥1 fully active drug in the optimized background regimen for eligibility, may limit the interpretation of comparisons at week 24. However, the aggregate clinical and resistance data support the choice of 50 mg twice daily as the appropriate DTG dose to be further evaluated in patients with INI-resistant virus.

In conclusion, during a period of functional monotherapy in subjects with limited treatment options and RAL-resistant HIV-1, DTG 50 mg twice daily provided substantial antiretroviral activity that was sustained through 24 weeks after optimization, when possible, of background therapy. As with other antiretroviral drugs, durability of response is more likely when there is support from background drug activity. Integrase resistance emergence during therapy occurred in <15% of all subjects, and the mutations that emerged were previously described RAL-associated mutations. As yet there is no in vivo evidence of emergence of novel mutations that result in a substantial decrease in DTG susceptibility. Because of the improved efficacy, good tolerability, and similar safety noted for DTG 50 mg twice daily, compared with DTG 50 mg once daily, the 50 mg twice-daily dosage is being evaluated in a larger, ongoing phase III study (VIKING-3) in a heavily treated HIV-1 infected population harboring RAL- or EVG-resistant virus.
